# Plaque Rupture Complications in Murine Atherosclerotic Vein Grafts Can Be Prevented by TIMP-1 Overexpression

**DOI:** 10.1371/journal.pone.0047134

**Published:** 2012-10-11

**Authors:** Margreet R. de Vries, Hans W. M. Niessen, Clemens W. G. M. Löwik, Jaap F. Hamming, J. Wouter Jukema, Paul H. A. Quax

**Affiliations:** 1 Einthoven Laboratory for Experimental Vascular Medicine, Leiden University Medical Center, Leiden, The Netherlands; 2 Department of Surgery, Leiden University Medical Center, Leiden, The Netherlands; 3 Department of Pathology and Cardiac Surgery, ICaR-VU, VU University Medical Center, Amsterdam, The Netherlands; 4 Department of Endocrinology, Leiden University Medical Center, Leiden, The Netherlands; 5 Department of Cardiology, Leiden University Medical Center, Leiden, The Netherlands; Heart Center Munich, Germany

## Abstract

The current study describes the incidence and phenotype of plaque rupture complications in murine vein grafts. Since matrix metalloproteinases (MMPs) are highly involved in atherosclerotic plaque vulnerability and plaque rupture, we hypothesized that this model can be validated by overexpression of the MMP inhibitor TIMP-1. First we studied 47 vein grafts in hypercholesterolemic ApoE3*Leiden mice for the incidence of plaque complications. In 79% of these grafts, extensive lesions with plaque rupture complications like dissections, intraplaque hemorrhages or erosions with intramural thrombi were found. Next, *in vivo* Near-InfraRed-Fluorescence imaging demonstrated that electroporation mediated TIMP-1-overexpression reduced local MMP activity in vein grafts by 73% (p<0.01). This led to a 40% reduction in lesion-size after 28d (p = 0.01) and a more stable lesion phenotype with significant more smooth muscle cells (135%), collagen (47%) and significant less macrophages (44%) and fibrin (55%) than controls. More importantly, lesions in the TIMP-1 group showed a 90% reduction of plaque complications (10/18 of control mice showed plaque complications versus 1/18 in TIMP-1 treated mice). Murine vein grafts are a relevant spontaneous model to study plaque stability and subsequent hemorrhagic complications, resulting in plaque instability. Moreover, inhibition of MMPs by TIMP-1-overexpression resulted in decreased plaque progression, increased stabilization and decreased plaque rupture complications in murine vein grafts.

## Introduction

Atherosclerosis and subsequent plaque complications are still a major cause of morbidity. In acute coronary syndrome, plaque rupture accounts for 75% of the deaths and plaque erosion causes the residual 25% [1]. Numerous factors such as endothelial activation, inflammation, cholesterol influx, necrotic core expansion, and cell death contribute to plaque instability and plaque rupture [2]. Furthermore, intraplaque angiogenesis contributes to plaque growth and unstable plaques by enhancing leukocyte recruitment and accumulation of cholesterol and platelets in the plaque [3], [4].

Essential in plaque instability are matrix metalloproteinases (MMPs), a group of zinc-containing endopeptidases, which degrade collagen and other extracellular matrix (ECM) components in the vessel wall [5]. A large repertoire of MMPs is found in atherosclerotic plaques [6]. The proteolytic activity of MMPs is regulated by tissue inhibitors of matrix metalloproteinases (TIMPs) which are also present in the vessel wall [7]. Due to the broad activity of MMPs in plaque development, interference in their regulation is an attractive strategy to decrease plaque formation and increase stability.

Although several atherosclerotic mouse models display features of intraplaque haemorrhage [8], [9] there is still need for mouse models of spontaneous atherothrombosis, the major cause of clinical manifestations. In the present study we use the model of vein graft interpositioning [10]–[12] as a model to study plaque instability and subsequent plaque complications.

Clinically, vein grafts are used to bypass an obstructed artery due to atherosclerosis. However, up to 40% of the vein grafts need revision within 18 months due to thrombosis, intimal hyperplasia and accelerated atherosclerosis [13], [14]. In murine vein grafts atherosclerotic lesions show features of unstable plaque phenotypes as observed in patients. Recently, we demonstrated that MMPs are highly expressed in these lesions [15]. The present study describes a detailed characterization of plaque instability and plaque rupture complications observed in vein graft lesions in hypercholesterolemic ApoE3Leiden mice. Subsequently the model was validated by studying the effect of systemic TIMP-1 overexpression on lesion progression and plaque rupture complications in this model.

**Figure 1:**
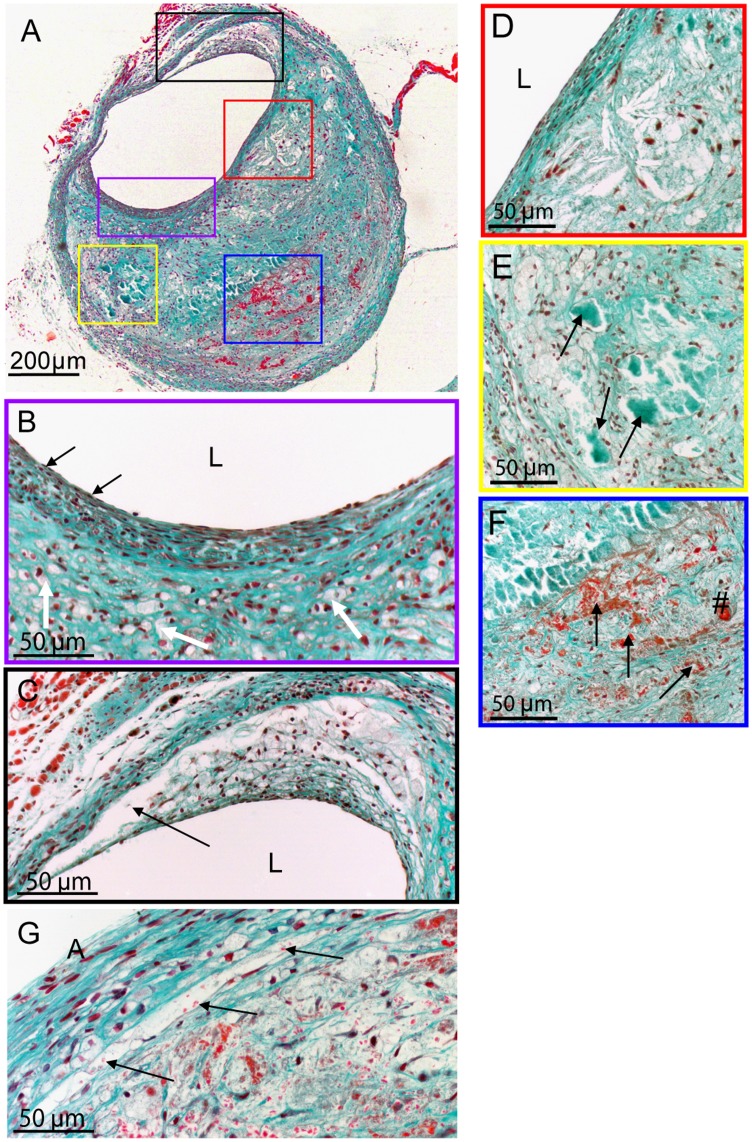
Vein graft lesion showing complex morphology including plaque rupture complications. **A**; Cross-section stained with a Masson trichrome staining of a vein graft in an ApoE3Leiden mouse 28 days after surgery. Coloured squares depict location of photographs B–G. L represents lumen and A; adventitia **B**; Part of the lesion showing foamcells (white arrows) and SMC’s, with a smooth muscle cell rich cap underneath an intact endothelial layer (black arrows; endothelial cells) **C**; Foamcell rich area including the start of a dissection. **D**; A small necrotic core with cholesterol clefts and foam cells can be seen with a SMC rich layer and endothelial cells on top. **E**; Calcification rich area (arrows) near the outer layer of the vessel wall. **F**; Area with extravasated erythrocytes (arrows) and neovascularization (#). **G**; Detailed photograph of dissection and extravasated erythrocytes 150 µm upstream in the lesion (arrows; erythrocytes stuck in the dissection).

## Methods

### Ethics Statement

This study was performed in compliance with Dutch government guidelines and all animal experiments were approved by the animal welfare committee of the Leiden University Medical Center.

### Animals

Male ApoE3Leiden mice (bred in our own colony), 10–16 weeks old, were fed a hypercholsterolemic diet (ABdiets, Woerden, The Netherlands) from 3 weeks prior to surgery until sacrifice. This resulted in plasma cholesterol levels between 12 and 24 mmol/l ((Roche Diagnostics, kit 1489437, Almere, The Netherlands). Before electroporation and surgery, mice were anesthetized with midazolam (5 mg/kg, Roche Diagnostics), medetomidine (0.5 mg/kg, Orion, Espoo, Finland) and fentanyl (0.05 mg/kg, Janssen Pharmaceutica, Diegum, Belgium). After the procedures the mice were antagonized with atipamezol (2.5 mg/kg, Orion) and fluminasenil (0.5 mg/kg Fresenius Kabi, Schelle, Belgium). Buprenorphine (0.1 mg/kg, MSD Animal Health, Boxmeer The Netherlands) was given after surgeries to relieve pain.

**Figure 2:**
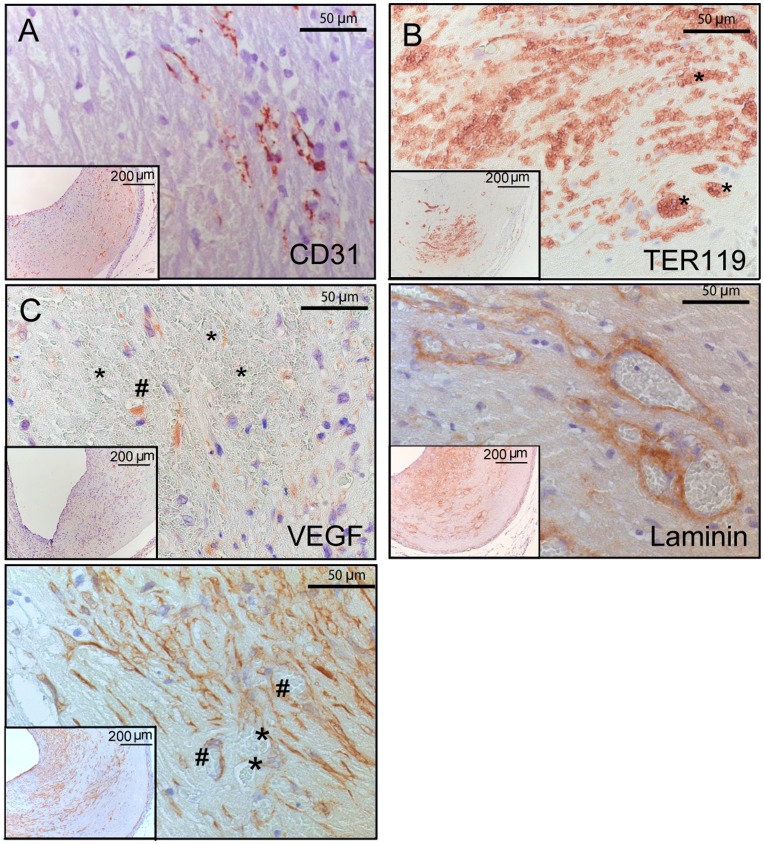
Vein graft lesions show clear neovascularization. **A**; Endothelial cell staining (CD31) of neovessels in a vein graft lesion. **B**; Erythrocytes are clearly visible in (*) and outside neovessels indicating that leaky vessels are present. **C**; VEGF staining of a vein graft with extravasated erythrocytes (*), a VEGF positive neovessel is depicted with #. **D**; Basement membrane staining with antibodies directed to laminin. Most neovessels in vein grafts stain positive. **E:** SMC staining of a vein graft.^ #^ marks SMC positive neovessels and * depicts neovessels lacking pericytes. These neovessels without pericytes are frequently found in regions with extravasated erythrocytes. Insets are 10x magnifications of vein grafts.

**Figure 3:**
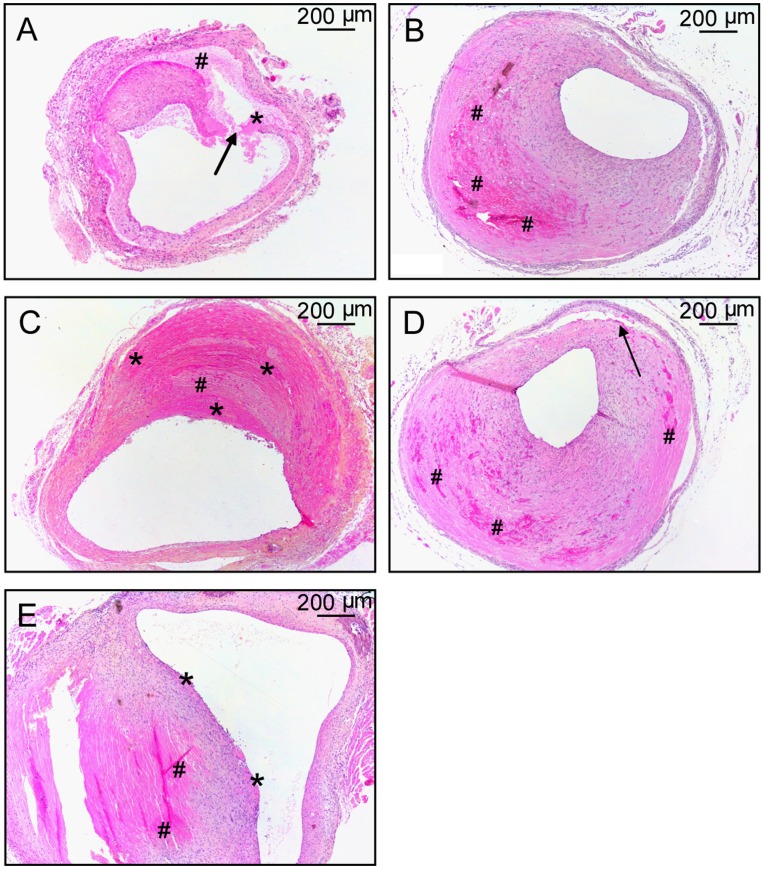
Divers forms of plaque rupture complications can be seen in vein graft lesions. **A**; Vein graft with a dissection (arrow) starting at the lumen of the vessel wall with small fibrin depositions (*) and erythrocytes (#) in the clearly visible gap. **B**; Vein graft lesion with leaky vessels and extravasated erythrocytes (#) **C;** Vein graft showing massive intramural thrombi consisting of layers of fibrin (*) and diffuse erythrocytes (#) with erosion extending to the outer part of the vessel wall **D**; Vein graft showing combined leaky vessels with extravasated erythrocytes (#) and dissection in the outer part of the vessel wall (black arrow). **E**; Vein graft lesion with leaky vessels showing extravasation of erythrocytes (#) and erosion with small mural thrombi (*).

### Luciferase and TIMP Overexpression

Overexpression of the TIMPs was achieved by electroporation after intramuscular injection of a pCDNA3.1 plasmid encoding for human TIMP-1 or TIMP-3 [16] and Luciferase as a control, one day before surgery. The plasmid DNA preparation and procedure of electroporation was performed as described previously [15].

**Figure 4:**
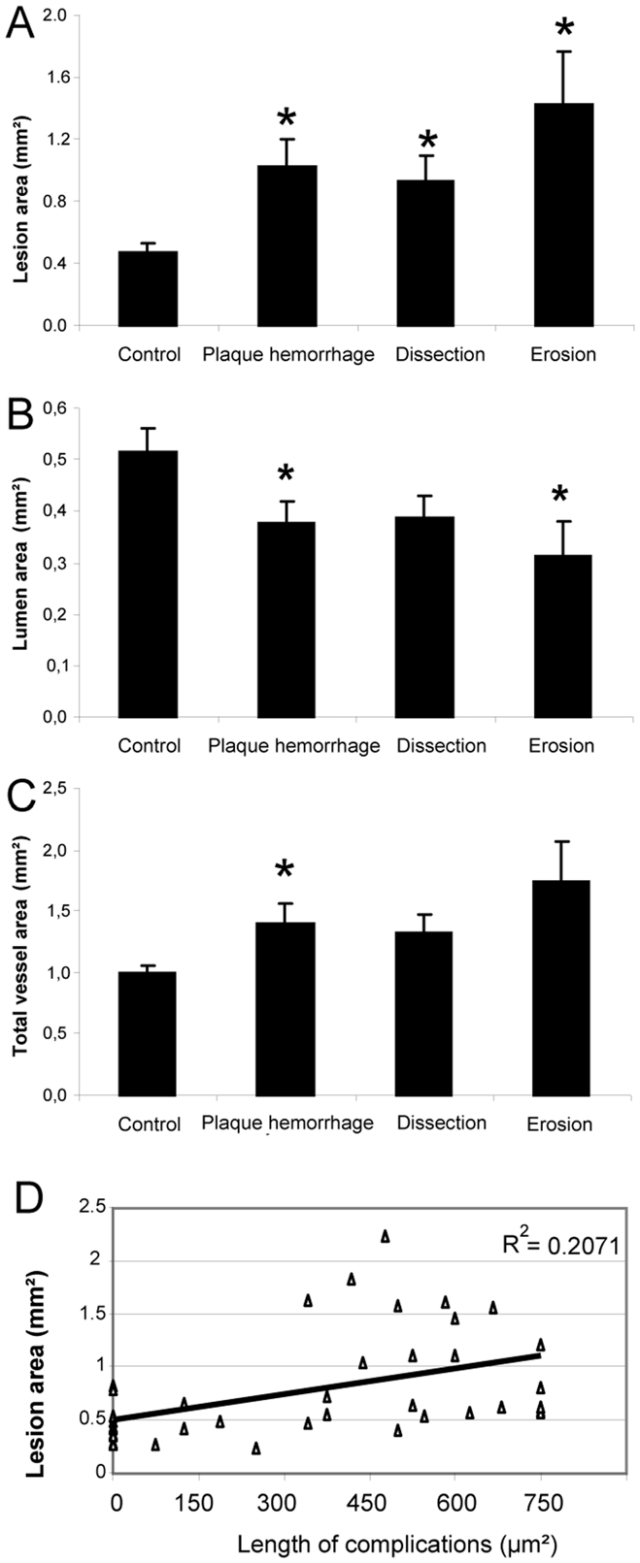
Quantification of vein grafts without complications (Control), and vein grafts with complications, namely plaque hemorrhage, dissections or erosions (n = 10/group). **A**; Vessel wall area measurements **B**; Quantification of lumen area **C**; Total vessel area (combined lumen and vessel wall area, as a measure for outward remodeling) **D**; Correlation between the vessel wall area and the length of the plaque rupture complications.

### Vein Graft Model

Vein graft surgery was performed by donor caval vein interpositioning in the carotid artery of recipient mice as described before [12]. In brief, thoracal caval veins from donor littermates were harvested. In recipients, the right carotid artery was dissected and cut in the middle. The artery was everted around the cuffs that were placed at both ends of the artery and ligated with 8.0 sutures. The caval vein was sleeved over the two cuffs, and ligated. Animals were sacrificed after 28 days, after 3 minutes of *in vivo* perfusion-fixation, vein grafts were harvested and fixed in 4% formaldehyde, dehydrated and paraffin-embedded for histology [17], [18].

**Figure 5:**
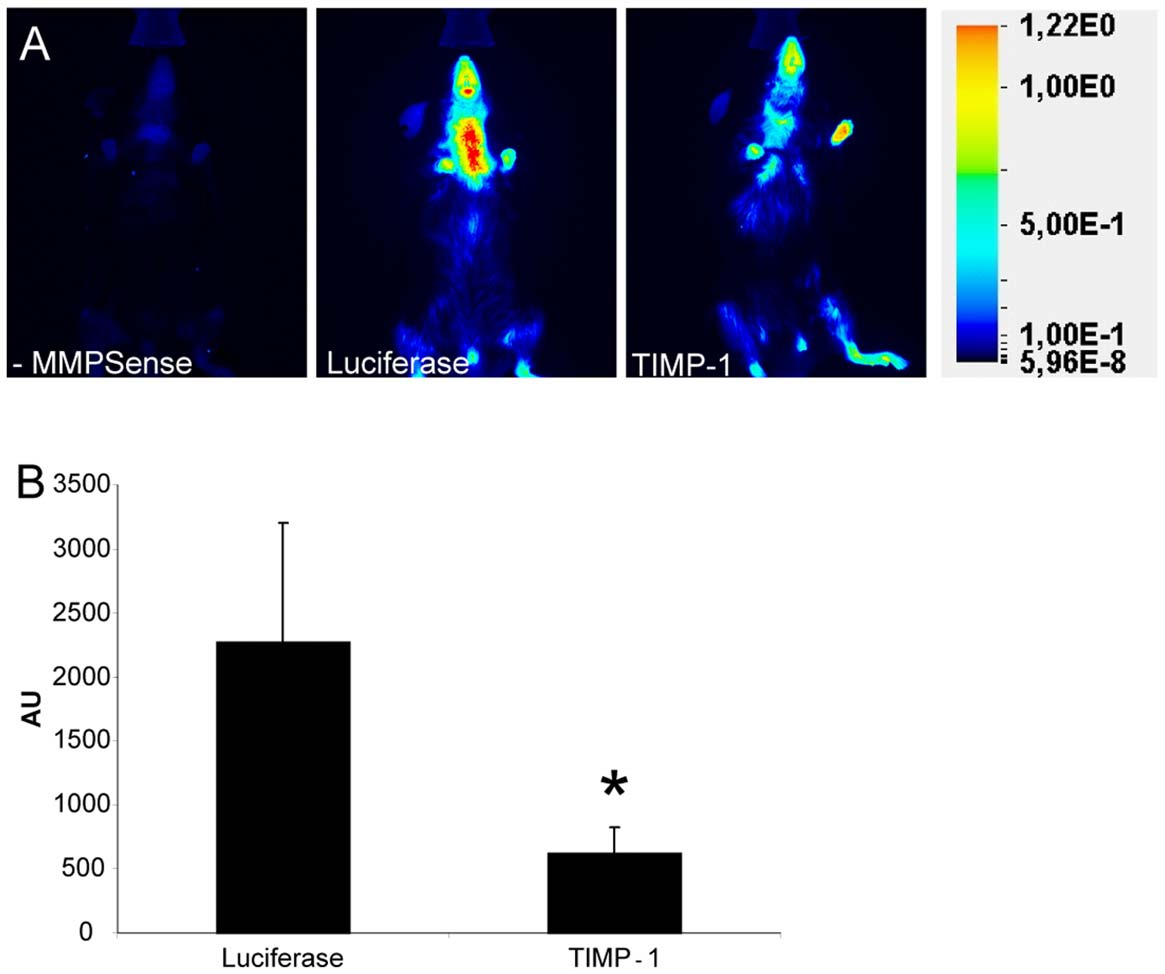
In vivo MMP activity in mice A; Representative Near-InfraRed-Fluorescence images of enzymatic MMP activity in mice. No signal was detected in mice without MMPSense. **B**; Quantification of the MMP activity in the vein graft region (n = 5/group).

### Histological and Immunohistochemical Assessment of Vein Grafts

Paraffin embedded cross sections were stained with hematoxylin-phloxine-saffron (HPS), Masson’s Trichrome stain and Picrosirius red (collagen). Antibodies against CD31 (endothelial cells) and MAC3 (macrophages) were purchased from Pharmingen (Heidelberg, Germany). Anti-SMC Actin (Sigma-Aldrich, St. Louis, MO, USA) was used to detect SMCs and pericytes. TER119 (erythrocytes) and anti-VEGF were purchased from Santa Cruz (CA, USA). Furthermore, Anti-laminin (Abcam, Cambridge, UK) and anti-fibrin (Quickzyme, Leiden, The Netherlands) were used. For each antibody, isotype-matched antibodies were used as negative controls and positive staining was absent in these sections (data not shown).

**Figure 6:**
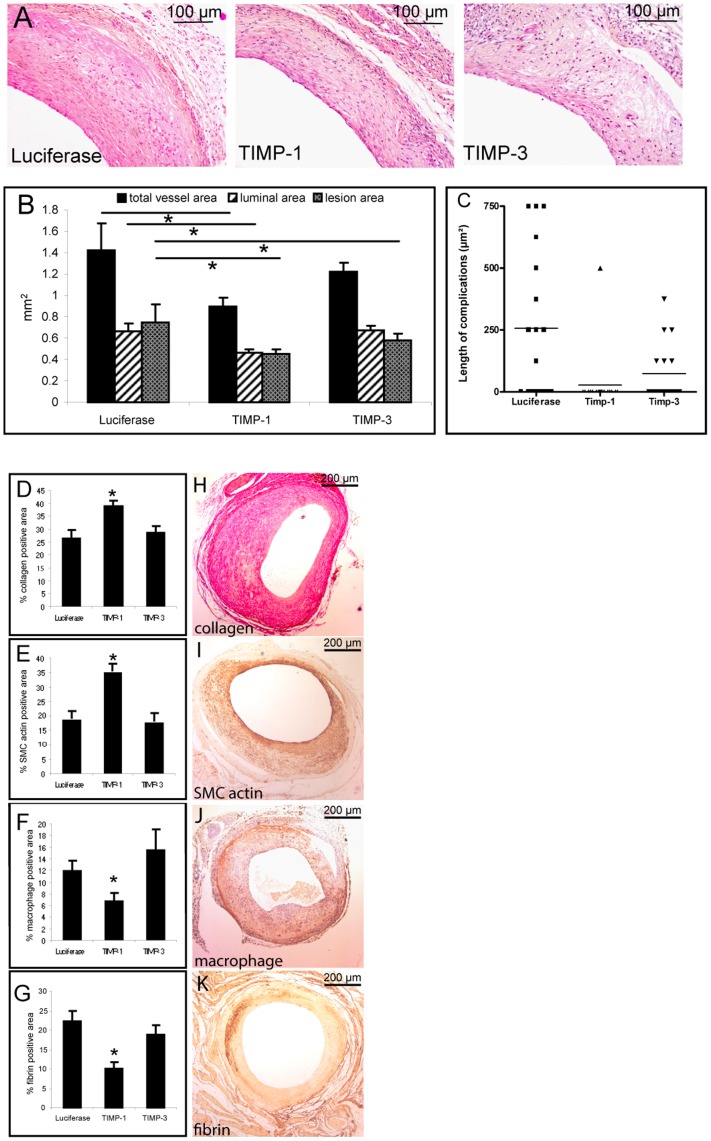
Quantitative measurements on vein graft lesions in mice that overexpress Luciferase, TIMP-1 or TIMP-3. **A**; Representative cross-sections of vein grafts in mice 28 days after surgery (Hematoxilin-Phloxine-Saffron staining). **B**; Quantitative measurements of total vessel area, luminal area and lesion area **C**; Graph showing the length of the plaque rupture complications. **D**; Quantitative measurement of percentage collagen in vein grafts in Luciferase, TIMP-1 or TIMP-3 overexpressing mice. **E–G**; Quantitative measurement of percentage SMC actin, macrophages and fibrin **H–K;** Typical examples for all (immuno)histochemical stainings.

### Morphologic Analyses of Vein Graft Rupture Complications

Vein graft rupture complications were scored in 47 mice. Lesions where erythrocytes were found adjacent to neovessels were regarded as lesions with plaque hemorrhage (hem). A dissection (diss) was defined as a connection between the lumen and the part of the vessel wall underneath the adventitia filled with fibrin and erythrocytes. In case fibrin was found at the luminal side underneath a denudated endothelial layer, coinciding with erythrocytes and neutrophils, we defined this structure as erosion with intramural thrombosis (er).

### Morphometric Analysis of Vein Grafts

Morphometric analysis of vein grafts harvested after 28d was performed using image analysis software (Qwin, Leica, Wetzlar, Germany). For each mouse six equally spaced (150 µm apart) cross-sections were used to determine lesion size and occurrence of plaque complications over a total vein graft length of 750 µm. Since elastic laminae do not exist in these grafts of venous origin, we analysed the putative vessel wall area (or lesion area) by measuring total vessel area (the area of the vessel wall within the adventitia and the lumen area. Next the lesion area was calculated (total vessel area – lumen area). (Immuno)histochemical staining was quantified by computer assisted analysis (Qwin, Leica). For this immuno-positive areas in vein grafts were expressed as percentage of the lesion area.

### *In vivo* NIRF Imaging

For Near-InfraRed-Fluorescence (NIRF) imaging experiments, MMPSense 680 (VisEN Medical) was used. Endogenous MMP-2,-7,-9,-12 and -13 can activate MMPSense *in vivo*, resulting in a fluorescent signal. Four nmol MMPSense was injected i.v. 20h before imaging (n = 5 mice/group). Images were acquired 28 days after surgery using a Pearl Imager (LI-COR Biosciences) and analysed using manufacturer’s software. Regions of interest (ROIs) of the vein areas were selected from equivalent-sized areas. ROIs were quantified for mean pixel values as shown previously [19].

### Statistical Analysis

All data are presented as mean ± SEM. Statistical analysis was performed using SPSS 17.0 for Windows. To determine statistical significance overall comparisons were made using the non-parametric Kruskal-Wallis test. In case of significance, each group was separately compared to the control group using the Mann-Whitney test. Probability-values <0.05 were regarded significant.

## Results

### Vein Graft Lesion Morphology

The vein graft wall of hypercholesterolemic ApoE3Leiden mice increases from a few cell layers at start of engraftment to a massive thickened vessel wall 28 days post-surgery. In vein grafts the lumen often was found eccentric, (figure 1A) probably due to flow differences in the curvature of the graft [20]. Surgical handling and dilatation of the vein graft result in de-endothelialisation. Vein grafts are repopulated to an almost intact endothelium (black arrows figure 1B and Figure S1A (CD31 staining)) within 28 days. The vein graft lesion consists of a dense network of ECM and smooth muscle cells (SMC). Circular orientated SMC were frequently seen close to the lumen suggesting a cap-like organization (white arrows, figure 1B and Figure S1B (SMC actin staining). Accumulations of lipid-laden foam cells were primarily found underneath the cap (Figure S1C (MAC-3 staining). Interestingly, plaque rupture complication in the form of a dissection from the lumen to the outer lesion was seen frequently in these vein grafts (Figure 1C and G). Foci of macrophages, foam cells, small necrotic cores and cholesterol crystals were found throughout the whole vessel wall, but most often near the luminal side (figure 1D). Amorphous calcifications were mostly found in the medial part and outer vessel wall area of the graft (figure 1E and Figure S1D (Von Kossa staining). In addition, cartilage, calcified cartilage and (ectopic) bone formation were found in these lesions, as shown previously [12]. In addition to the plaque rupture complications, unique for this mouse model is that neovessels were found throughout the vein graft wall. Neovessel showed a preference for the medial and outer part. The newly formed vessels were often leaky as erythrocytes were found adjacent to these neovessels (Figure 1F). The outer side of the graft include the adventitia and connective tissue, consisting of fibroblasts, ECM and neovessels, as depicted in figure 1G.

### Vein Graft Plaque Neovasculature

To focus more specifically at the neovessels in the vein grafts specific immunohistochemical stainings were performed. Neovessels in the vein graft wall are most likely formed through angiogenesis and consist of CD31 positive endothelial cells (figure 2A). In a substantial number of vein grafts, a considerable amount of erythrocytes could be found in the ECM, adjacent to the neovessels (figure 2B and Figure S2) suggesting leakiness. Expression of Vascular Endothelial Growth Factor (VEGF) was analysed. VEGF not only induces angiogenesis and endothelial cell proliferation but can also increase vascular permeability [21]. VEGF (figure 2C) then not only was found adjacent to the neovessels but also seems to co-localize with macrophages and SMC, both known VEGF producers. As leaky vessels can be the result of abnormal maturation of neovessels [22], we subsequently stained for laminin, a marker for basement membranes and with anti–SMC-actin, to detect pericytes and SMCs surrounding the neovessels, both essential components for proper maturation. Almost all most neovessels had intact basement membranes (figure 2D). In contrast, not all neovessels showed efficient pericyte coverage, especially neovessels in regions of erythrocyte extravasation (figure 2E).

### Frequency and Appearance of Plaque Rupture Complications

In murine vein grafts, plaque rupture complications were frequently found, which were subsequently quantified. In 79% of these mice plaque complications were observed. 16 out of 47 (34%) vein grafts showed lesions with dissections (Figure 3A). Another 34% showed extravasations of erythrocytes within the lesion (Figure 3B). Finally 30% of the vein grafts showed erosion with intramural thrombosis. Occlusive luminal thrombi were rarely found instead intramural thrombi with dispersed erythrocytes and neutrophils were found (Figure 3C).

Strikingly, in some of these vein grafts combinations of plaque hemorrhage and dissection (11%, Figure 3D) or plaque hemorrhage and erosion (9%, Figure 3E) were found. This demonstrates that in these vein grafts complex unstable atherosclerotic lesions were induced.

### Lesion Size of Vein Grafts and Plaque Rupture Complications

To study whether a correlation exists between the degree of vein graft lesion size and the occurrence of plaque rupture complications, lesion area was quantified in vein grafts without complications (ctr), mice displaying dissections (diss), erosion with intramural thrombosis (er) or plaque hemorrhage (hem) (n = 10/group).

Mice with plaque hemorrhage or dissections showed, compared to control mice, a 2-fold increase in lesion area (ctr; 0.474±0.052 mm^2^ vs. hem; 1.027±0.173 mm^2^ p = 0.004 vs. diss; 0.934±0.155 mm^2^ p = 0.005, figure 4A) whereas mice with erosion showed a 3-fold increase (1.423±0.336 mm^2^ p = 0.045). In contrast, luminal area was significantly decreased in vein grafts with plaque hemorrhage and erosion (ctr; 0.516±0.044 mm^2^ vs. hem; 0.377±0.043 mm^2^ p = 0.033 vs. er; 0.313±0.086 mm^2^ p = 0.021). The luminal area in vein grafts with dissections was also decreased compared with controls, although this did not reach significance (0.386±0.042 mm^2^ p = 0.064), figure 4B. Total vessel area (luminal area and lesion area combined), as a measure for remodeling, was only significantly different in the group with plaque hemorrhage (ctr; 0.990±0.062 mm^2^ vs. hem; 1.404±0.153 mm^2^ p = 0.028 vs. diss; 1.321±0.148 mm^2^ p = 0.111 vs. er; 1.736±0.326 mm^2^ p = 0.064) as shown in figure 4C. Finally, it was analysed whether a correlation existed between the length of the vein graft segment that displayed plaque rupture complication and the lesion area and indeed a weak positive correlation was found (R^2^∶0.207).

### TIMP-1 Overexpression Results in Decreased MMP Activity in Vein Grafts In Vivo

Since MMPs play a major role in atherosclerosis, the effects on MMP activity in the vein graft in vivo were analysed using NIRF imaging technique. To prove a causal role of MMP activity in vein graft lesions, TIMP-1 was overexpressed in mice receiving a venous graft. As depicted in figure 5A, MMP activity was detected in the neck region of Luciferase overexpressing control mice, thus the area of the vein graft. In the TIMP-1 treated group (TIMP-1) a significant reduction of 73% in MMP activity was found (616±205AU vs. 2272±932AU; p = 0.009, figure 5B) compared to the Luciferase group (Luc).

### Morphological Analysis of Vein Grafts after TIMP Overexpression

TIMP-1 overexpression resulted in a decrease in total vessel area of 36% (Luc 1.42±0.25 mm^2^ vs. TIMP-1 0.90±0.08 mm^2^ p = 0.003), a decrease in lumen area of 30% (Luc 0.66±0.08 mm^2^ vs. TIMP-1 0.46±0.04 mm^2^ p = 0.005) and a profound decrease in lesion area of 40% (Luc 0.75±0.17 mm^2^ vs. TIMP-1 0.45±0.05 mm^2^ p = 0.010) compared to the Luciferase control group (figure 6A&B) 28 days post-surgery. Next, we analysed the occurrence of plaque rupture complications. In the Luciferase group 10 out of 18 mice showed plaque complications of all three forms. Strikingly only 1 of the 18 TIMP-1 group showed a plaque rupture complication, namely plaque hemorrhage, which is significantly different compared with the Luciferase group (p = 0.001).

To determine whether the observed difference in frequency of plaque rupture complications correlates specifically with TIMP1 expression, or is a result of a decrease in lesion area, a comparable experiment was performed using an alternative way to reduce MMP activity, by TIMP-3 overexpression. TIMP-3 is a MMP inhibitor that has a slightly different substrate specificity than TIMP-1, but with similar potency to block MMP activity [7]. In these TIMP-3 overexpressing mice (n = 17) a reduction in lesion area of 33% compared to control mice was found (p = 0.035), with no significant difference between the TIMP-1 and TIMP-3 group (p = 0.790). When compared to the control group no differences in total vessel area (1.42±0.25 mm^2^ vs.1.22±0.09 mm^2^ p = 0.266) and lumen area (0.66±0.08 mm^2^ vs. 0.67±0.05 mm^2^ p = 1.000) were found (figure 6B). Subsequently, plaque rupture complication events were analysed. In the TIMP-3 group 6 out of 17 mice showed plaque rupture complications. This was not significantly different compared to the control group (p = 0.238) whereas it was significantly increased compared to the TIMP-1 group (p = 0.030). Moreover, the length of the segments displaying complications in the TIMP-1 group clearly was smaller than the length of the segments with rupture complications detected in the controls (p = 0.002) and TIMP-3 group (p = 0.045). Albeit no differences were found between control and TIMP-3 group (p = 0.071) (Figure 6C).

Next the effects on vein graft lesion composition were assessed. TIMP-1 overexpression resulted in a significant enhancement of stability improving factors: increased collagen content (TIMP-1∶34.6±2.5% vs. Control: 18.5±2.1% vs. TIMP-3∶17.4±2.5% p = 0.007, figure 6D) and smooth muscle cells presence, figure 6E (SMC positive area of the lesion: TIMP-1∶38.6±2.4% vs. Control: 26.2±3.3% vs. TIMP-3∶28.6±2.5% p = 0.003). Moreover, TIMP-1-overexpression resulted in a major decrease in factors known for their contribution to plaque destabilization, namely macrophages, figure 6F (MAC-3 positive area of the lesion: TIMP-1∶6.7±1.4% vs. Control: 11.9±1.7% vs. TIMP-3∶15.5±3.5% p = 0.03) and fibrin, figure 6G (Fibrin positive area: TIMP-1∶9.9±1.7% vs. Control: 22.2±2.7% vs. TIMP-3∶18.8±2.3% p = 0.01). No significant differences in plaque stabilizing and destabilizing factors were seen between control and TIMP-3 overexpressing mice.

## Discussion

Vein grafts in hypercholesterolemic ApoE3Leiden mice show complex lesions which resemble, to a large extent, native (human) atherosclerotic plaques. Although the accelerated formation of atherosclerotic lesion in this vein graft model in hypercholesterolemic ApoE3Leiden mice is first described in 2002 [12], detailed analysis of the histology of the lesion now for the first time reveals several features that mimic important aspects of plaque instability and rupture, including intraplaque hemorrhage and angiogenesis. These novel aspects of the atherosclerotic vein graft lesions make this model an interesting model for studying crucial aspects of plaque instability, intraplaque hemorrhage and plaque rupture. Plaque rupture complications such as dissection, erosion with intramural thrombosis and leaky vessels (as depicted in Figures 1 and 3) as well as intraplaque neovascularization (Figure 2) shown here, are rarely seen in other murine atherosclerotic models.

One of the rare models for spontaneous plaque rupture and intraplaque hemorrhage in the mouse is the rupture of lesion described to occur in the brachiocephalic artery of ApoE deficient mice [9], [23]. In this brachiocephalic artery after 48–54 weeks lesions develop that show features of intraplaque hemorrhage, and in mice fed a pro-atherogenic diet even plaque rupture can be observed [9]. However, it takes up to 14 months to develop these lesions. Moreover, the qualification of these lesions as being ruptured plaques is strongly debated [24], since the observed intraplaque hemorrhage seems to be less complex, lacking clear atherothrombotic events [24]. The fact that in the vein graft model the lesions may form within four weeks, with a range of plaque destabilisation features occurring, underscores the advantages of the model for studying several aspects of plaque destabilization. Importantly, in 79% of these vein grafts, features of intraplaque hemorrhage and plaque rupture occur spontaneously. Even combined plaque complications were found, illustrating the complexity of these lesions. Validation of the plaque rupture model by overexpressing TIMP-1 showed that plaque instability could be modulated towards a more stable plaque phenotype with less plaque rupture complications.

Although plaque rupture was clearly present, occlusive luminal thrombi, the major cause of vascular clinical deaths, were rarely seen in this vein graft model. This can be in part caused by the faster coagulation of thrombi in mice due to higher activity of endogenous coagulation inhibitors compared to humans [25] since mice have lower levels of thrombin-activated fibrinolysis inhibitor (TAFI) and plasminogen activator inhibitor-1 (PAI-1) [26], [27].

A weak positive correlation (R^2^ = 0.207) was found between lesion area and the percentage of plaque complications. This suggests that plaque complications are likely not only the result of increasing plaque size. In this respect, the plaque phenotype is probably more important. This is also seen in human lesion where most rupture-prone plaques are unstable but not per se stenotic [1]. Plaque rupture, defined as a disruption of a (thin) fibrous cap over a lipid core with direct contact of the lipid core with the blood in the lumen resulting in a occlusive luminal thrombosis, was not observed in the vein graft model [28]. This can be attributed by the fact that vein graft plaques consist of heterogeneous distribution of SMC, foamcells and small necrotic cores [12], [29] compared to the large necrotic core with an overlying cap which are prominent in human atherosclerosis. Although no plaque rupture in the classical definition as such was observed, dissections were frequently found in the vein graft lesions. Denudation of the endothelium resulted in thrombosis into the vessel wall with profound areas of fibrin and erythrocytes. As these thrombi mostly intruded a large part of the vessel wall, this resulted in an enormous increase in vessel wall area. Inflammatory cells were scarce in these lesions except for neutrophils, which is comparable to what Farb et al. found in human plaque erosion [30]. Another interesting finding in this study was the occurrence of neovessels that in majority displayed a non-mature phenotype. Besides a decrease in lumen area and outward remodeling of the vein graft, lesions with leaky vessels also displayed enhanced vessel wall thickening and infiltration of inflammatory cells around the neovessels. This concurs with previous studies on the role of leaky vessels in human plaque stability [3], [22].

MMPs play an important role in the atherosclerotic plaque development, but more importantly, in the regulation of plaque instability as demonstrated by knockdown or overexpression studies in mice [31]–[37]. Moreover, Rouis et al. demonstrated that adenoviral overexpression of TIMP-1 resulted in a decrease in atherosclerotic lesion area and more stable lesions in ApoE deficient mice [38]. Johnson et al showed no effect of adenoviral TIMP-1 overexpression on brachiocephalic artery plaque size, but suggested a change towards a more stable plaque phenotype due to an increase in elastin content in the plaque [39].

We have previously demonstrated expression of MMPs in mouse vein grafts in vivo [15], [40]. Furthermore, we and others have demonstrated that TIMP-1 overexpression can inhibit atherosclerotic lesion formation [15], [38]. Here we studied the effects of TIMP-1 on plaque stability and plaque rupture. TIMP-1 overexpression resulted in a significant reduction of in vivo MMP-activity of 73% indicating activity of one or a combination of the MMPs -2,-7,-9,-12 or -13 in the vein graft. More importantly, overexpression of TIMP-1 resulted in reduced vein graft lesion size, reduced macrophage and fibrin content, increased collagen and smooth muscle cell content and ultimately resulted in a 90% reduction in plaque rupture complications. Also the severity of the complications, as scored by the length of the complications, was significant lower in the TIMP-1 treated group. Although overexpression of TIMP-3 also resulted in a significant decrease in lesion area, it had in contrast to TIMP-1 no significant effect on plaque phenotype. TIMP-1 differs from TIMP–3 by being a poor inhibitor of the membrane type MMPs (MT-MMPs). Although MT-MMP1 is known to be involved in the breakdown of collagen and activation of pro-MMP-2 and -13 [41], inhibition of the other MMPs by TIMP-1 is apparently more important to favour a stable phenotype after overexpression.

In conclusion, this study shows that vein grafts in ApoE3Leiden mice generate lesions with spontaneous rupture complications that can be used as a unique animal model to study mechanisms of atherothrombotic lesions. Intriguingly, TIMP-1 overexpression, but not TIMP-3, resulted in a more stable phenotype and most importantly a decrease in incidence and severity of plaque complications.

## Supporting Information

Figure S1 **Immunohistochemical stainings demonstrate specific phenotypic features of vein grafts.** **A**. 28 days after vein graft surgery an intact endothelium is present in most vein grafts. **B**. SMCs are found distributed throughout the whole vein graft wall, a denser region of SMC can be found near the lumen displaying a cap-like phenotype. **C**. lipid loaden macrophages can be seen in all segments of the vein graft but especially near the cap. **D**. Areas of calcification can be found in most vein grafts, depicted over here as black staining.
(TIF)

Figure S2 **Neovessels are present in vein graft lesions.** These neovessels (**A**) are filled with erythrocytes (**B**) which are also found outside the neovessels as a result of leaky vessels. This can be clearly seen in the overlay with false colors (**C**) of the consecutive sections of A and B.
(TIF)

Information S1 **Methods for immunohistochemistry used in this study.**
(DOCX)
