# Supplementary figures and images for: Plaque Rupture Complications in Murine Atherosclerotic Vein Grafts Can Be Prevented by TIMP-1 Overexpression
Source: PLoS One. 2012 Oct 11;7(10):e47134. doi: 10.1371/journal.pone.0047134 (PMC3469549; doi:10.1371/journal.pone.0047134)

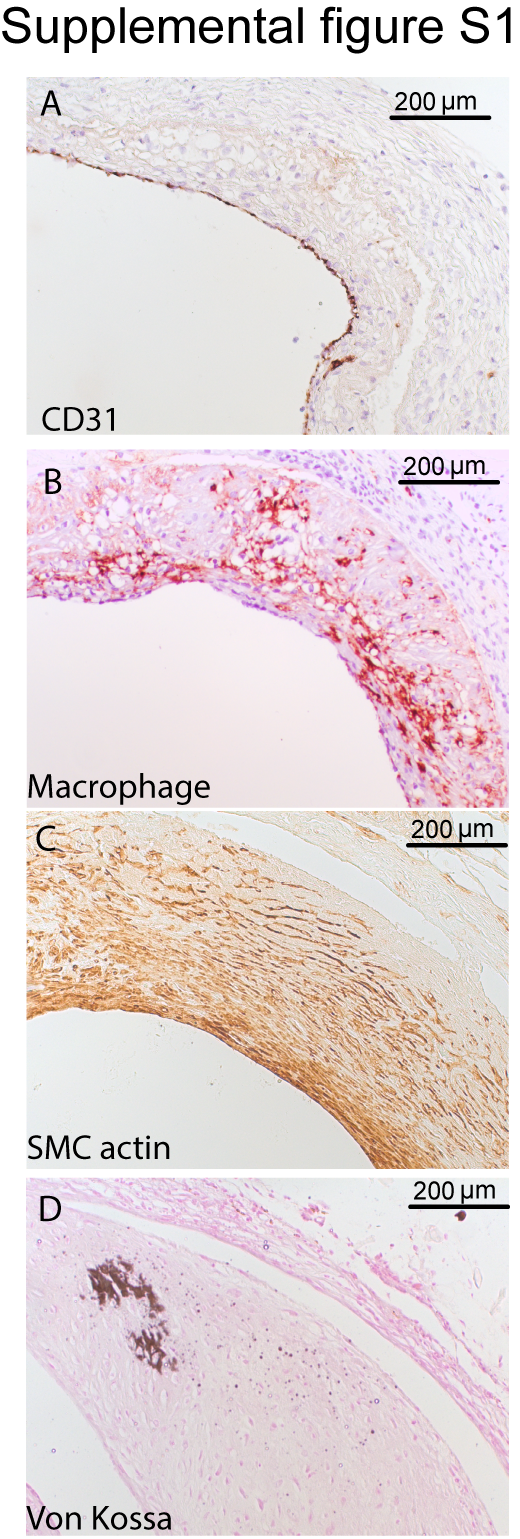

Supplement: Figure S1 — Immunohistochemical stainings demonstrate specific phenotypic features of vein grafts. A. 28 days after vein graft surgery an intact endothelium is present in most vein grafts. B. SMCs are found distributed throughout the whole vein graft wall, a denser region of SMC can be found near the lumen displaying a cap-like phenotype. C. lipid loaden macrophages can be seen in all segments of the vein graft but especially near the cap. D. Areas of calcification can be found in most vein grafts, depicted over here as black staining. (TIF) [file pone.0047134.s001.tif]

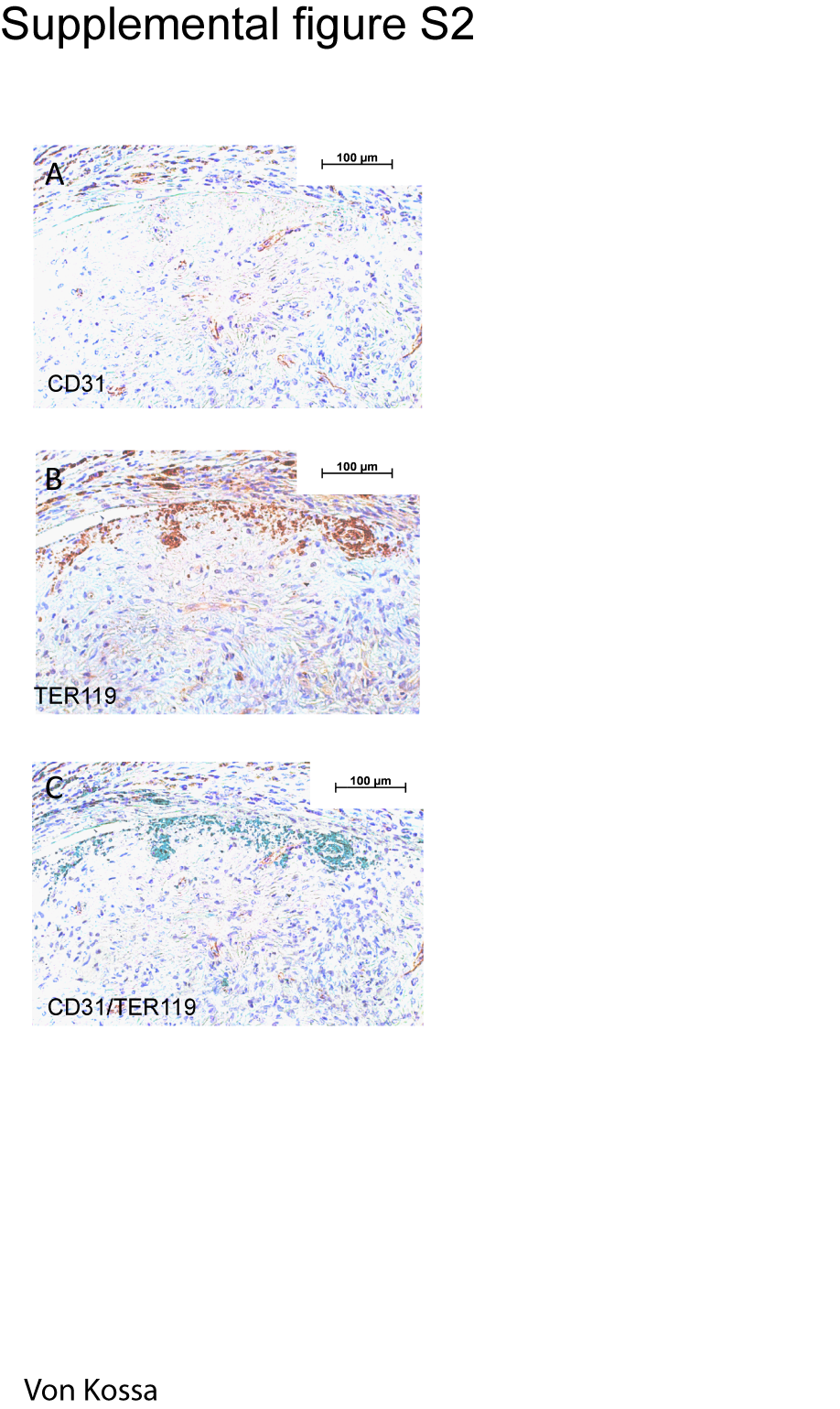

Supplement: Figure S2 — Neovessels are present in vein graft lesions. These neovessels (A) are filled with erythrocytes (B) which are also found outside the neovessels as a result of leaky vessels. This can be clearly seen in the overlay with false colors (C) of the consecutive sections of A and B. (TIF) [file pone.0047134.s002.tif]
