# Supplementary material for: Plaque Rupture Complications in Murine Atherosclerotic Vein Grafts Can Be Prevented by TIMP-1 Overexpression
Source: PLoS One. 2012 Oct 11;7(10):e47134. doi: 10.1371/journal.pone.0047134 (PMC3469549; doi:10.1371/journal.pone.0047134)
Supplement: Information S1 — Methods for immunohistochemistry used in this study. (DOCX) [file pone.0047134.s003.docx]

**S3 Supporting information**

**Methods**

***Histological and immunohistochemical assessment of vein grafts:***

Cross sections of vein grafts were routinely stained with hematoxylin-phloxine-saffron (HPS) and Masson’s Trichrome stain. For detection of calcifications, a von Kossa staining was used, as described previously [1]. Antibodies against CD31 and MAC3 (BD-Pharmingen) were used to detect endothelial cells and macrophages, respectively. Anti-SMC Actin (1A4, Sigma) was used to detect smooth muscle cells and TER119 (Santa Cruz) for erythrocytes. For each antibody, isotype-matched antibodies were used as negative controls and positive staining was absent in these sections (data not shown).

**Results**

The vein graft wall of hypercholesterolemic ApoE3Leiden mice increases from a few cell layers at start of engraftment to a massive thickened vessel wall 28 days post-surgery. Surgical handling and dilatation of the vein graft result in de-endothelialisation. Vein grafts are repopulated to an almost intact endothelium (supplemental Figure S1A (CD31 staining)) within 28 days. The vein graft lesion consists of a dense network of ECM and smooth muscle cells (SMC). Circular orientated SMC were frequently seen close to the lumen suggesting a cap-like organization (supplemental Figure S1B (SMC actin staining)). Accumulations of lipid-laden foam cells were found primarily underneath the cap (supplemental Figure S1C (MAC-3 staining)). Amorphous calcifications were mostly found in the medial part and outer vessel wall area of the graft, as shown in supplemental Figure S1D (Von Kossa staining). In addition, cartilage, calcified cartilage and (ectopic) bone formation were found in these lesions, as shown previously [1] . The lesions in the vein grafts also contain neovessels (supplemental Figure S2A) which are filled with erythrocytes (supplemental Figure S2B). Some of these neovessels are leaky since erythrocytes can be demonstrated outside these neovessels, as shown in supplemental Figure S2C. This figure shows an overlay (with false colors) of the consecutive sections of supplemental figure S2A and S2B.

Reference List

1. Lardenoye JH, de Vries MR, Lowik CW, Xu Q, Dhore CR, et al. (2002) Accelerated atherosclerosis and calcification in vein grafts: a study in APOE*3 Leiden transgenic mice. Circ Res 91: 577-584.
